# 

**Published:** 1946

**Authors:** 


Vol. LXIII. No. 226. SUMMER, 1946.

### The Bristol Medico-Chirurgical Journal

PUBLISHED UNDER THE AUSPICES OF

THE BRISTOL MEDICO-CHIRURGICAL SOCIETY.

*Editor:* J. A. NIXON, C.M.G., M.D., F.R.C.P.

WITH WHOM ARE ASSOCIATED

A. RENDLE SHORT, M.D., B.S., B.Sc., F.R.C.S.

W. MELVILLE CAPPER, M.B., B.S., F.R.C.S.

G. E. F. SUTTON, M.C., M.D., B.S., M.R.C.P.

E. WATSON-WILLIAMS, M.C., Ch.M., F.R.C.S., *Assistant Editor.*

Price Four Shillings net.

